# Development of nested polymerase chain reaction-based diagnosis of duck enteritis virus and detection of DNA polymerase gene from non-descriptive duck breeds of West Bengal, India

**DOI:** 10.14202/vetworld.2017.336-341

**Published:** 2017-03-21

**Authors:** Partha Sarathi Mandal, Sunit Kumar Mukhopadhayay, Saktipada Pradhan, Samiran Mondal, Chandrakanta Jana, Nimai Chandra Patra, Rabindra Nath Hansda

**Affiliations:** 1 Block Animal Health Centre, Gorubathan, Darjeeling, West Bengal, India; 2 Department of Veterinary Pathology, West Bengal University of Animal & Fishery Sciences, Kolkata - 700 037, West Bengal, India; 3 Division of Pathology, ICAR - Indian Veterinary Research Institute, Mukteswar Campus, Mukteswar, Uttarakhand, India

**Keywords:** chorioallantoic membrane histopathology, nested polymerase chain reaction, polymerase chain reaction

## Abstract

**Aim:**

The study was undertaken to detect the clinical signs, postmortem lesions of embryonated duck plague (DP) infected eggs, and histopathological changes of chorioallantoic membrane (CAM) in non-descriptive ducks of West Bengal with special reference to standardize nested polymerase chain reaction (PCR).

**Materials and Methods:**

After postmortem of suspected carcasses, samples were collected for virus isolation and identification through specific pathogen free (Khaki Campbell) embryonated duck eggs. PCR was also done as confirmatory test after doing postmortem of duck embryos. DP specific nested PCR was standardized for better confirmation of the disease. Sensitivity of nested primers was also tested for DP virus.

**Results:**

Gross, postmortem and histopathological changes were prominent in dead embryos. First set of primer was able to detect 602 bp fragments of DNA polymerase gene of duck enteritis virus from infected CAM. Subsequently, a DP specific nested PCR which was very much sensitive for very small amount of viral genome was successfully standardized. After NCBI blast nucleotide sequence of nested PCR product (Accession No. HG425076) showed homology with the sequences data available in GenBank.

**Conclusion:**

The study concludes that PCR assay is very much helpful to diagnose DP disease and developed nested PCR is a double confirmatory diagnostic tool for DP.

## Introduction

Duck virus enteritis (DVE) or duck plague (DP) is one of the most devastating diseases of duck, which facilitates huge economic losses every year in West Bengal. This disease is caused by anatid herpesvirus-1 under subfamily of Alphaherpesvirinae of family *Herpesviridae* [1]. The disease was first recorded from West Bengal [2] in India and subsequently from Tamil Nadu [3], Kerala [4], and again from West Bengal [5]. According to Animal Disease Surveillance Report of Government of West Bengal (2013), some DP prone zones of Hooghly, Burdwan, Purulia, Dakshin Dinajpur, and North-24 Parganas districts were selected for this study, where high mortality and morbidity was observed in previous years from non-descriptive duck breeds. The disease produced significant economic loss as a result of high mortality in non-descriptive ducks of West Bengal. Previously, various studies were reported on duck enteritis virus (DEV) isolation and identification of various recognized duck breeds [6-8], but no report has been found till now on non-descriptive duck breeds of West Bengal.

Various serodiagnostic methods such as enzyme-linked immunosorbent assay [9], indirect enzyme-linked immunosorbent assay [10], counter-current immunoelectrophoresis [11], passive hemagglutination [6], and latex agglutination test have been described throughout the world to diagnose DP on recognised duck breeds, but these tests are not so suitable for getting genetic information about the pathogen. Hence, polymerase chain reaction (PCR) was chosen to diagnose the DP from non-descriptive ducks. Standardization of nested PCR assay for DEV diagnosis is a double confirmatory tool which was not tested previously for DP and also gives genetic information about the viral nucleic acid.

This type of nested PCR assay was able to detect very minute amount of virus concentration [12], and the phylogenetic analysis of viral DNA was helpful to identify the similarity with other Alphaherpesvirinae. This study will help in easy, early confirmation of the disease and the gene sequence data can give us knowledge over the change of virulence of the virus which has great importance on ineffectiveness of general vaccination against field outbreaks.

## Materials and Methods

### Ethical approval

The study was ethically appoved by Institutional Animal Ethics Committee, Faculty of Veterinary and Animal Science, West Bengal University of Animal and fishery Sciences, before start of experiment.

### Sample preparation and virus isolation

Postmortem of suspected DP carcasses (Table-1, n=56) were conducted after taking of a proper history, and 2 g of liver and spleen were taken aseptically for virus isolation from each sample of the outbreaks. Samples were homogenized in saline water containing 2000 IU/ml penicillin and 200 mcg/ml streptomycin. Sterile phosphate buffer solution (pH 7.4) was added with the homogenized tissue to prepare a 20% (W/V) tissue suspension and centrifuged at 3000 rpm for 15 min [13] and filtrated through 0.45 µm membrane filter. The suspension was subsequently incubated at 37°C for 45 min.

**Table-1:** Details of sample collection from field outbreaks in West Bengal.

Place of out breaks	Postmortem done	Collected sample
Hooghly (Dhaniakhali block)	16	Liver, spleen
Burdwan (Kalna-1 block)	11	Liver, spleen
Purulia (Puncha block)	9	Liver, spleen
Dakshin Dinajpur (Banshihari block)	8	Liver, spleen
North 24 Parganas (Amdanga block)	12	Liver, spleen
Total (n)	56	

A total number of 60 specific pathogen free Khaki Campbell duck eggs were taken and divided into six groups (according to sample collection) where one group served as control. Each 10-day-old embryonated duck egg of five experimental groups were inoculated with antibiotic-treated suspension (0.2 ml), and the same amount of phosphate-buffered saline was inoculated in control group through chorioallantoic membrane (CAM) route [14]. Consequently, three passages were done where in each passages 10-day-old embryonated eggs were used for inoculation. Thereafter, mortality of the embryos were monitored daily for next 10 days. CAM and allantoic fluid (AF) from last passages of all groups were separately collected and stored at −20°C for PCR (CAM and AF) and in 10% formalin for histopathology (CAM). Samples for histopathology were processed through paraffin embedded technique and sections were cut at 4 µm thickness and then cut sections were stained with hematoxylin and eosin [15].

### Genome preparation

DNA was extracted and purified from (n=56) infected CAM and AF as per protocol of DAN Sure^®^ Tissue Mini Kit, Genetix Biotech Asia (P) Ltd. Extracted DNA concentration was calculated with the help of Bio-photometer (Eppendorf, Germany) by taking reading at 260 and 280 nm and concentration of the samples were ranged from 0.251 to 0.50 µg/µl.

### Primers

First set of primers (Table-2) were designed for identification of DEV on the basis of the partial nucleotide sequence of DNA polymerase gene (GenBank Accession No. AF064639) of a DEV vaccine strain [12]. Based on Genbank Accession No. JQ655152 of Indian DEV isolate, the primers for secondary PCR were designed.

**Table-2:** Sequences of DP primers used for this research work.

Primers	5ˊ-3ˊ sequence
UL-F UL-R	GGCTGGTATGCGTGACAT GTATTGGT TTCTGAGTTGGC
DP-F DP-R	GATGTAGACGAAGGCGGGTA TACGCTGTCCACGTCAGTTT

DP=Duck plague

### PCR

First set of primers (UL-F and UL-R) were used to develop a 602 bp fragment [12,16] from extracted DNA of infected CAM and AF through PCR assay with some alteration. Volume of total reaction mixture (50 µl) was composed of 2 µl (40-45 ng concentration) of DEV genomic DNA, 25 µl of ×2 PCR master mix (Fermentas, USA), 21 µl nuclease free water (Fermentas, USA), and 1 µl (10 pmol/µl cocentration) of each forward (UL-F) and reverse (UL-R) primers. PCR was performed in a thermocycler (Mastercycler, Eppendorf, Hamburg, Germany) using the following protocol: Denaturation at 94°C for 4 min (one cycle), 30 cycles of 94°C for 1 min (denaturation), 55°C for 50 s (annealing), 72°C for 2 min (extension) which was followed by final extension at 72°C for 7 min. The amplified product of PCR assay was checked by agarose gel electrophoresis through ultraviolet (UV) transilluminator (Bio-Rad, USA).

### Standardization of secondary/nested PCR

The amplified product (Hooghly isolates) by the first set of primers (UL-F and UL-R) were diluted with nuclease free water to make the final concentration 40 ng/µl and was taken as template for standardization of secondary (Nested) PCR assay. Each 50 µl volume of reaction mixture was composed of 25 µl ×2 master mix (Fermentas, USA), 3 µl of gnomic DNA, 20 µl nuclease free water (Fermentas, USA), and 1 µl (10 pmol/µl cocentration) of each forward (UL-F) and reverse (UL-R) primer. PCR amplification was done using the following protocol: Denaturation at 94°C for 5 min (one cycle), 35 cycles at 94°C for 1 min (denaturation), 35 cycles at 50°C for 45 s (annealing), 35 cycles at 72°C for 1 min (elongation), one cycle 72°C for 7 min (final elongation). The amplified product of nested PCR assay was checked by 1% agarose gel electrophoresis through UV transilluminator (Bio-Rad, USA).

### Sensitivity of secondary/nested PCR

A serial 10-fold dilution set was prepared in 10 sterile Eppendorf tubes from diluted (40 ng/µl) primary PCR product and 3 µl from each dilution was added to reaction tubes as template, containing second sets (DP-F and DP-R) of primers for PCR sensitivity test [12]. PCR was performed as described earlier in standardization of nested PCR. PCR product was evaluated in 1% agarose gel and examined under UV transilluminator system (Bio-Rad, USA).

### Nucleic acid sequence study

For the further experiment, nested PCR sample (Hoogly isolates) was sent to Xcelris Laboratory, Ahmedabad for sequencing in an automated DNA sequencer. The nucleotide sequence generated in this study has been deposited in EMBL (nested PCR product) database to obtain accession number. Data analysis and multiple alignments were performed using ClustalX [17] and MEGA6 software [18]. The sequence was compared with GenBank database using the BLASTn. The evolutionary distances were computed using the p-distance method [19]. The confidence values of the internal rods were calculated by performing 1000 bootstrap analyses [20]. Phylogenetic tree for nucleotide sequences was drawn using the neighbor-joining method [21].

## Results

### Clinical and histopathological changes in embryo

**Figure-1:**
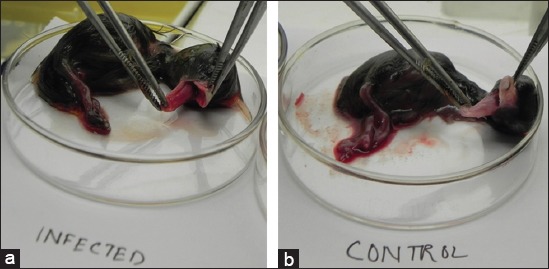
(a) Reddish color of infected duck embryo along with wide spread petechiae throughout the skin. (b) Control group of embryo does not showed reddish skin coat).

**Figure-2:**
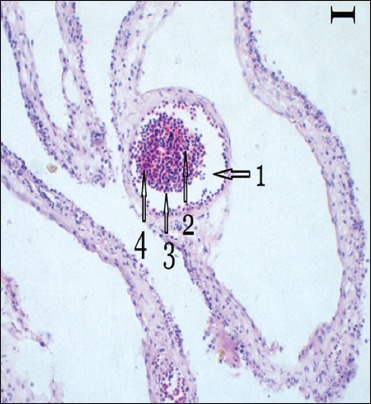
Chorioallantoic membrane histopathology shows infiltration of mononuclear cells (arrow head 2 and 3) and heterofils (arrow head 4) with round inflammatory zones (arrow head 1) (H and E, 200× µm).

Death of embryos started first from Hoogly inoculates (4^th^ day of post inoculation) and last from North-24 Parganas (8-10^th^ days of post inoculation). During the postmortem of inoculated egg embryos, except Purulia isolates other four showed changes like as reddish skin with petechiae (Figure-1a), widespread congestion and hemorrhage on CAM, enlargement of the liver, petechial hemorrhage and necrotic foci on spleen. Microscopically infected CAM showed enormous hemorrhagic lesions, round inflammatory zones with infiltration of mononuclear cells and heterophils (Figure-2). In post mortem examination of embryo, Purulia isolates do not show any change (Figure-1b) as like the control group, in comparison with other four isolates.

### Analysis of primary PCR

**Figure-3:**
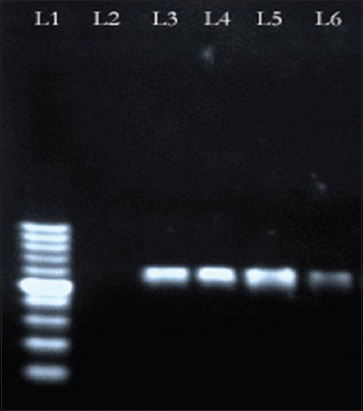
Polymerase chain reaction (PCR) result shows 100 bp ladder in Lane 1; Lane 2 - Negative isolates of Purulia and 602 bp amplified products in Lane 3 - Hooghly; Lane 4 - Burdwan; Lane 5 - Dakshin Dinajpur; Lane 6 - North-24 Parganas.

The primers (UL-F and UL-R) were able to amplify 602 bp fragment (Figure-3) of DNA polymerase gene from 47 infected CAM and AF of four different (Hooghly, Burdwan, Dakshin Dinajpur and North-24 Parganas) isolates. The isolates of Purulia showed negative result (Lane-2 of Figure-3) in PCR.

### Development of nested PCR

**Figure-4:**
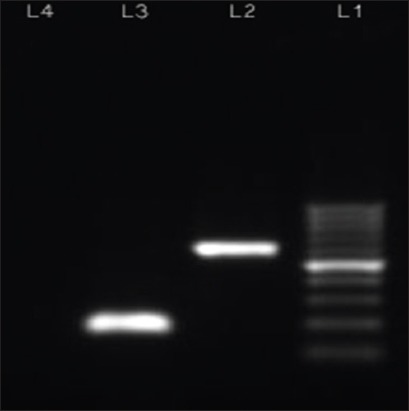
Photograph shows Lane 1 - 100 bp ladder; Lane 2 - 602 pb primary polymerase chain reaction amplicon; Lane 3 - 189 bp nested polymerase chain reaction amplicon; Lane 4 - Negative control.

Both primary and secondary (nested) PCR products were analyzed by agarose gel electrophoresis where a clear 602 bp band for the primary PCR products (Lane-2 of Figure-4) and a clear 189 bp band (Lane-3 of Figure-4) for nested PCR products were present.

### Sensitivity of nested PCR

**Figure-5:**
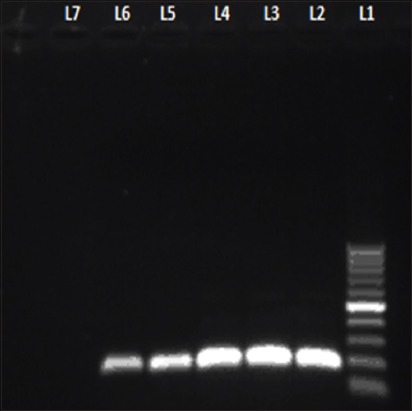
Sensitivity testing shows Lane 1 - 100 bp ladder; Lane 2-6 - Amplicon of 189 bp by second set primers after 10-fold serial dilution of diluted primary polymerase chain reaction product; Lane-7 - Negative dilution.

The primers of the second set (DP-F and DP-R) were able to amplify 189 bp products (Figure-5) up to 10^−5^ dilution (0.4 pg) of DNA concentration, but these primers were not able to amplify DEV genome beyond this 10^−5^ fold dilution (Lane-7, Figure-5) of template.

### Sequence study and phylogenetic analysis

**Figure-6:**
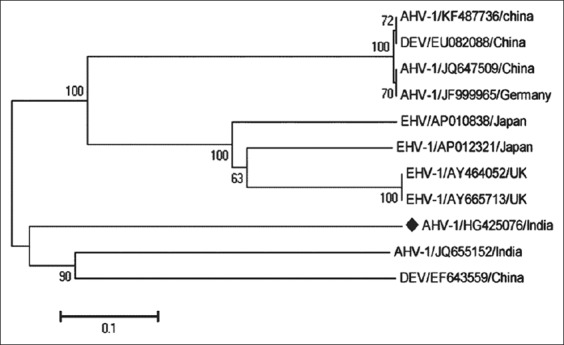
Phylogenetic tree of duck enteritis virus (DEV) isolates HG425076 with other published DEV sequences available in GenBank.

Nucleotide sequence analysis of obtained accession number HG425076 showed some polymorphism and one gape in respect to other published sequences. NCBI BLAST of the nucleotide sequence (Accession No. HG425076) showed 97% homology (Table-3) with other anatid herpes virus-1 sequences. In phylogenetic analysis (Figure-6) nested sequence (Accession No. HG425076) clustered closely with JQ655152 of India and EF643559 of China. Rest of the sequences formed two different groups, one for anatid herpes virus-1 and another for equine herpes virus.

**Table-3:** Homology of DNA polymerase gene (Accession No. HG425076) sequence of DEV with other herpes virus gene sequences available in GenBank.

Accession No.	Country	Homology (%) of nucleotide sequence HG425076 with other herpes virus
KF487736	China	313/313 (97)
JQ655152	India	313/313 (97)
JQ647509	China	313/313 (97)
JF999965	Germany	313/313 (97)
EU082088	China	313/313 (97)
EF643559	China	313/313 (97)

DEV=Duck enteritis virus

## Discussion

Death of embryos from 4^th^ day [2] to 10^th^ day of post inoculation confirms the different strength of severity of the viruses [7], isolated from various outbreaks except Purulia outbreak where embryos were alive. Reddish skin lesions with widespread petechiae, patches of congestion and hemorrhage on CAM of infected embryos correlate the disease with other previous studies [22]. Petechial hemorrhage and enlargement of the liver and necrotic foci on spleen [2,6] also indicate the outbreaks as DP. Our study focused down the histopathological changes of DEV-infected CAM first time which was enormous hemorrhage on CAM wall, round inflammatory zones with infiltration of mononuclear cells and heterophils. Redness or hemorrhagic lesions were not found in control and Purulia group of embryos. A product of 602 bp fragment of DNA polymerase gene [16] from infected CAM and AF of four different isolates indicates that PCR assay is a rapid and specific [12] diagnostic tool for DP disease. UL-F and UL-R primers were not able to develop positive bands in PCR from Purulia outbreaks which indicate that the reason of duck mortality in Purulia was not due to DEV.

Nested PCR gives us better confirmation about the DEV DNA polymerase gene amplicon as it involves two sets of primers, used in two successive runs of PCR, the second set intended to amplify a secondary target within the first run product and reduce non-specific binding in products due to the amplification of unexpected primer binding sites. The second set of primers (DP-F and DP-R) amplified down till 0.4 pg of DNA which indicates that second set primers are very much sensitive for detecting very small amount of DP viral genome material [12,23,24]. Hence, the nested PCR can be helpful for detecting DP viruses in subclinical, chronic cases or from reservoirs (carrier states) of the suspected flocks. Nested PCR assay for detecting of DP viral genome was not tested previously which we have also done first time in this study. Thus, the increased sensitivity of nested primers can be used to detect low-level DP virus shedding in waterfowl [12].

The polymorphism of nested sequence (HG425076) in respect to Accession No. JQ655152 indicates viral resistance against the host immunity and vaccination because mutation effects the binding characteristic of the receptors with antibodies [25,26]. Homology (97%) with other published GenBank sequence revealed that the amplified PCR product (189 bp) was of DNA polymerase gene of alphaherpesvirus origin [27]. The phylogenetic analysis of this sequence with other anatid herpesvirus and equine herpesvirus revealed three different clusters. Our reported sequence (HG425076) showed maximum similarities with anatid herpesviruses [28,29] like JQ655152 of India, EF643559 of China and less similarities with another nucleotide sequence, i.e., KF487736 of China, EU082088 of China, JQ647509 of China and JF999965 of Germany [27]. All equine herpesvirus nucleotides such as AP010838 of Japan, AP012321 of Japan, AY464052 of UK, and AY665713 of UK formed a different cluster which was distinct from anatid herpesvirus-1, indicates host specificity of the virus [28,30].

## Conclusions

Correlation of various data like field mortality history and symptoms, embryo inoculation test, PCR test confirmed the disease as DP of non-descriptive ducks of West Bengal. Besides this, development of nested PCR tool as double confirmatory test for DP infection and CAM histology is unique in this study.

## Authors’ Contributions

This study was a part of M.V.Sc. thesis of first author PSM under the guidance of SKM. SP, NCP and CJ helped during the trial and laboratory works. The article was drafted by PSM and revision was made by SM and RNH. All authors have read and approved the final manuscript.
